# Effect of TTN Mutations on Immune Microenvironment and Efficacy of Immunotherapy in Lung Adenocarcinoma Patients

**DOI:** 10.3389/fonc.2021.725292

**Published:** 2021-08-26

**Authors:** Zhe Wang, Chunguang Wang, Shengcheng Lin, Xin Yu

**Affiliations:** National Cancer Center/National Clinical Research Center for Cancer/Cancer Hospital & Shenzhen Hospital, Chinese Academy of Medical Sciences and Peking Union Medical College, Shenzhen, China

**Keywords:** TTN, lung adenocarcinoma, immune checkpoint inhibitors, tumor microenvironment, immunogenicity

## Abstract

Immune checkpoint inhibitors (ICIs) effectively treat lung adenocarcinoma (LUAD) with fewer side effects. However, for LUAD patients, the lack of predictive markers for ICIs makes their clinical benefits less than ideal. Despite reports suggesting that a TTN (titin) mutation plays an important role in immunotherapy of solid tumors and gastric cancer, the relationship between the TTN mutation and LUAD immunotherapy has not been determined. We collected a LUAD cohort with whole-exome sequencing (WES) and immunotherapy prognosis. The ICI cohort was used to explore the relationship between TTN mutation status and prognosis. Then, the Cancer Genome Atlas (TCGA)-LUAD and Chen-LUAD cohorts were downloaded from the cbioportal website. We also used CIBERSORT, gene-set enrichment analysis (GSEA), and single-sample GSEA (ssGSEA) to evaluate the proportion of immune cells and the degree of pathway activation in LUAD patients, respectively. DDR signaling pathways obtained from the Molecular Signatures Database (MSigDB), tumor mutation burden (TMB), and NAL were used to evaluate the immunogenicity of LUAD patients. In the ICI cohort, TTN-mutant (TTN-MT) had significantly longer overall survival (OS) than TTN-wildtype (TTN-WT) (P = 0.009). Univariate and multivariate COX models showed that TTN mutation status can independently predict immunotherapy prognosis. Notably, the results of tumor immune microenvironment (TIME) analysis showed that TTN-MT patients had inflammatory TIME, which showed enriched activated immune cells and higher immune scores. Immunogenicity analysis showed higher immunogenicity in TTN-MT patients, which indicated high levels of gene mutations in TMB, NAL, and DDR pathways. GSEA and ssGSEA results showed that TTN-MT was substantially enriched in chemokine secretion, inflammatory factor secretion, and antigen presentation. Some pathways related to immunosuppression and immune depletion were significantly downregulated. TTN-MT is associated with significantly prolonged OS in LUAD patients. Additionally, TTN-MT is related to high immunogenicity and inflammatory TIME, suggesting that TTN-MT may be a potential predictive marker for patients with LUAD to accept ICIs.

## Introduction

At present, lung cancer tumor malignancy presents with the highest rates of morbidity and mortality, while lung adenocarcinoma (LUAD) is the main subclass of primary lung cancer (about 85%) (1). LUAD has a long incubation period, mild early symptoms, and a high degree of malignancy. Usually, LUAD patients progress to the advanced stage at the time of diagnosis and miss the chance for treatment (2). With the development of various targeted drugs and their application in LUAD, the 5-year survival rate of LUAD patients has been somewhat improved, especially with immunotherapy drug applications such as the programmed cell death protein 1/programmed cell death protein ligand 1 (PD-1/PD-L1). However, there are new challenges, and the response rate of some patients to immunotherapy is not high (3, 4). Therefore, it is imperative to identify new biomarkers to predict the curative effect of immunotherapy.

Increasingly more studies have started looking for the predictive markers of ICIs, in an attempt to maximize the clinical benefits of immunotherapy for patients. The research shows that traits such as PD-L1 expression, TMB, microsatellite instability (MSI), DNA damage repair (DDR), tumor-infiltrating lymphocytes (TILs) may be predictors of patients’ responses to immunotherapy. However, the widespread implementation of these markers has numerous limitations. For example, the PD-L1 detection methods lack standardization and consistency, TMB is heterogeneous among different laboratories and platforms, only a small number of patients with lung cancer contain the MSI-H phenotype (< 1%) (5–9), and results are based on the next-generation sequencing (NGS) evaluation of different panels. These factors suggest that finding new markers to predict immunotherapy efficacy is an urgent problem to be solved in precision immunotherapy.

TTN is used to express myosin, which is connected by unstructured peptide sequences and is responsible for maintaining muscle tension (10), while actin is the third richest protein in muscle. Because the TTN gene contains a long sequence, any mutation in the TTN gene may lead to the dysfunction of myosin, leading to the abnormal growth of muscle fibers (10). Many previous studies have only focused on a TTN mutation associated with skeletal muscle dystrophy and familial hypertrophic cardiomyopathy (11). However, in recent years, increasingly more studies have started examining the relationship between a TTN mutation and the immunotherapy response of solid tumors (12, 13). Jia et al. revealed a significant positive correlation between objective response rate (ORR) and the TTN mutation frequency after receiving anti-PD-1/PD-L1/CTLA-4 monotherapy (P = 0.0015; R = 0.5796). They also found that the OS of TTN-mutant (TTN-MT) patients was significantly longer than that of TTN-wildtype (TTN-WT) patients in the immunotherapy cohort of melanoma (log-rank P = 0.0113) and observed a notable reduction in the risk of adverse reactions (13).

Additionally, Yang and his colleagues found that MUC4, MUC16, and TTN mutations were associated with the immune prognosis of gastric cancer in the immunotherapy cohort (12). With the development of clinical trials for LUAD immunotherapy, the relationship between TTN-MT and immunotherapy prognosis and LUAD response remains unclear. To explore the relationship between TTN-MT and the clinical prognosis of LUAD patients after receiving ICIs, we collected and analyzed a LUAD cohort following anti-PD-1/PD-L1/CTLA-4 monotherapy, a TCGA-LUAD cohort, and another published LUAD cohort. This paper attempts to explore the relationship between TTN mutations and immunotherapy within the TIME.

## Methods

### Collection of LUAD Cohort

We downloaded a LUAD cohort (ICIs-cohort) with ICIs-based treatment plans from the supplementary materials of a published study (12). The cohort’s treatment plan and sequencing method are detailed in this study. In the follow-up analysis, we used the mutation data (WES) and clinical data of this cohort. Additionally, we acquired RNA-seq and mutation data from the Genomic Data Commons (GDC; https://portal.gdc.cancer.Gov/) database of TCGA-LUAD cohort. Using cbioportal (http://www.cbioportal.org/), we downloaded another LUAD cohort (Chen-LUAD), which includes expression data and mutation data (WES) (14). The pathological diagnoses are detailed in the **Supplementary Methods**.

### Analysis of TIME and Immunogenicity

The relative proportion of 22 immune cells were evaluated using the CIBERSORT algorithm (15), which can be implemented in http://cibersort.stanford.edu/. The CIBERSORT algorithm analyzes the expression data in the TCGA-LUAD cohort according to LM22 (22 immune cells) signature and 1,000 permutations. Additionally, they play corresponding roles in the immune response (e.g., antigen processing and presentation, inflammatory response, chemokines, and immune exhaustion) obtained from the supplementary materials of published research (16, 17). TMB, NAL, and immune scores were obtained from a published study (17), while the DDR pathway gene set was obtained from the MsigDB database (18).

### Pathway Analysis

Based on the GSEA, we used the R package “limma” to analyze the difference between RNA-seq data of TCGA-LUAD and Chen-LUAD. TCGA-LUAD, Chen-LUAD, logFC, and ENTREZID were used as GSEA input files in the difference analysis (19). The R package “ClusterProfiler” was used to analyze the path sets of input data (rank logFC and ENTREZID) in the GO-BP, GO-CC, GO-MF, KEGG, and REACTOME databases (20). For the ssGSEA, we used the R package “GSVA” (21) to analyze RNA-seq data of TCGA-LUAD and Chen-LUAD, and the set of pathways used includes c2.cp.kegg.v7.1.Symbols.gmt, c2.cp.reactiome.v7.1.symbols.gmt, and c5.all.v7.1.symbols.gmt, which were obtained from the MsigDB database (18).

### Statistical Analysis

The Mann-Whitney-U test was used to compare the differences in continuous variables between two groups: TMB, NAL; the ratio of immune cell infiltration; and the expression of immune-related genes. Additionally, we used Fisher’s exact test to compare the differences in classification variables between the two groups, such as gene mutation frequency. The Kaplan-Meier (KM) method (22) was used to compare the difference in survival time between the two groups, while the log-rank P-value represents the statistical difference between the KM analyses. GSEA analysis used the P-value and Enrichment score to evaluate the statistical differences in the GSEA results. Next, we used the R package “limma” to compare the differences in related pathological pathways between the two groups.

## Results

### TTN-MT Is Related to the Prognosis of LUAD Patients Receiving Immunotherapy

**Figure 1:**
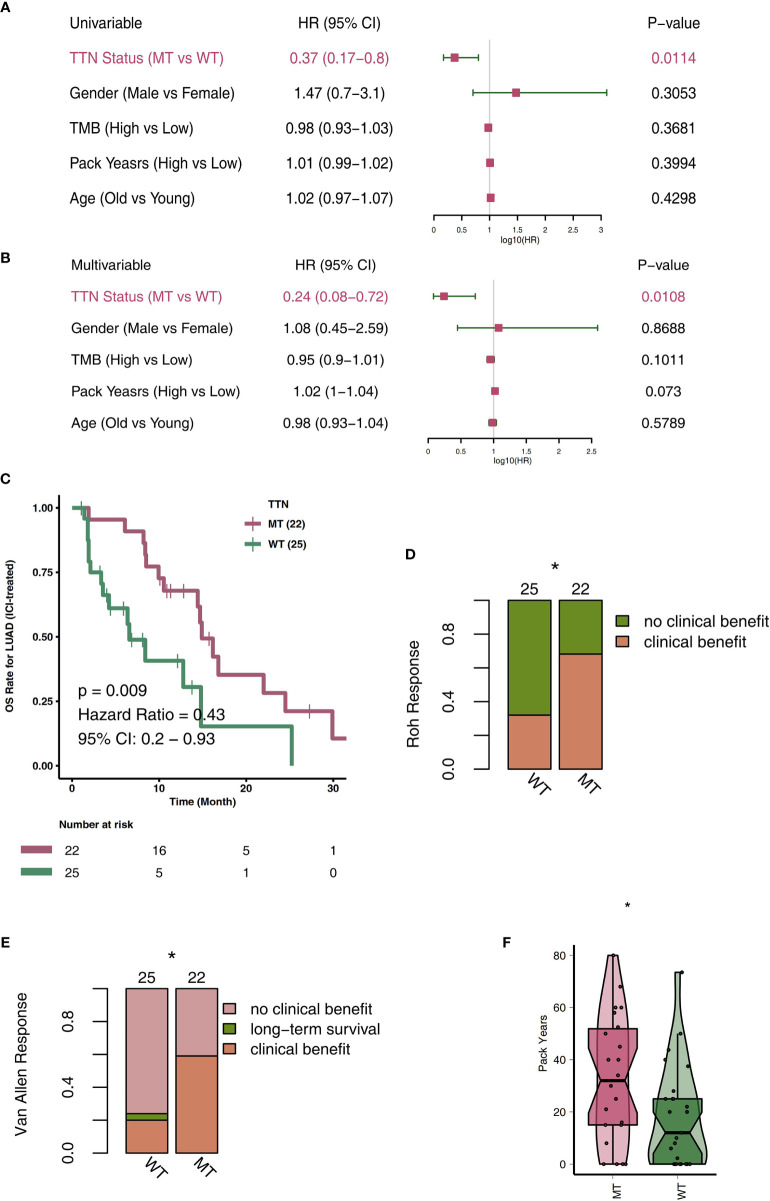
TTN-MT was associated with prolonged OS in LUAD patients responding to treatment with ICIs. Univariable Cox regression analysis **(A)** and multivariable Cox regression analysis in subgroups of TTN status, age, sex, TMB status, and pack years in the ICI cohort **(B)**. **(C)** Kaplan-Meier curves comparing the overall survival (OS) of patients with TTN-MT and patients with TTN-WT in the ICI-cohort. **(D)** TTN-MT was associated with clinical benefit (Roh response). **(E)** TTN-MT was associated with clinical benefit (Van Allen). **(F)** TTN-MT was associated with longer pack years. *P < 0.05.

To explore TTN-MT’s predictive role in the prognosis of LUAD patients after ICI therapy, we used a univariate COX regression model and multivariate COX regression model for the follow-up analyses. The two forest maps show the results of the univariate COX analysis and multivariate COX analysis, respectively. **Figure 1A** shows that only TTN mutations can be used to predict the prognosis of LUAD patients in the univariate COX regression model (P = 0.014; HR: 0.37). We found that other clinical characteristics (such as gender, age, pack years) and TMB, a known factor related to the immunotherapy prognosis, were not related to the prognosis of LUAD patients. **Figure 1B** shows that only the TTN mutation status can be used as an independent predictor of immunotherapy for LUAD patients in the multivariate COX regression model (P = 0.0108; HR: 0.24). Then, we used KM analysis to further reveal the role of TTN mutation in the OS time of LUAD patients. Compared with TTN-WT LUAD, patients with TTN-MT LUAD had significantly improved OS time [log-rank P = 0.009, HR = 0.43; 95% Cl: 0.2–0.93 (**Figure 1C**)]. Additionally, LUAD has a higher proportion of clinical benefits of immunotherapy than improved OS time, TTN-MT (**Figures 1D, E**). Additionally, we found that patients with TTN-MT LUAD had significantly increased pack years in comparison to TTN-WT LUAD (**Figure 1F**).

### Overview of Mutated Driver Genes in the TTN Mutation Status

**Figure 2:**
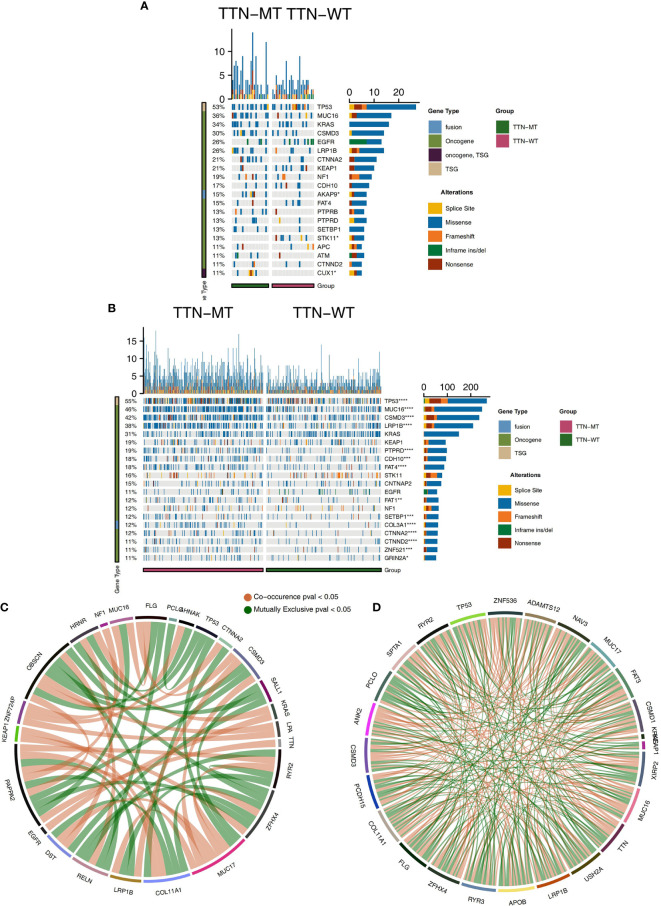
Genomic profiles of LUAD patients. The Top 20 mutated driver genes in the ICI-cohort **(A)** and the TCGA-LUAD cohort **(B)**. Ribbon plot showing the co-occurrence (or mutually exclusive relation) between pairs of mutated driver genes in the ICI-cohort **(C)** and the TCGA-LUAD cohort **(D)**. *P < 0.05; **P < 0.01; ***P < 0.001; ****P < 0.0001.

To explore the profiles of mutated driver genes in different TTN mutation statuses, we analyzed the 20 driver genes with the highest mutation frequency in LUAD patients in the ICI-cohort and TCGA cohorts, respectively. Most of the driver gene mutation types in the ICI cohort are mainly missense mutations [such as TP53, MUC16, KRAS, LRP1B, CTNNA2, KEAP1, NF1, CDH10, AKAP9, FAT4, PTPRB, PTPRD, SETBP1, STK11, ATM, and CTNND2 (**Figure 2A**)]. Furthermore, most of the ICI cohort’s top 20 driver genes with high mutation frequencies belong to an oncogene, and few belong to TSGs. Additionally, the mutation frequencies of AKAP9 (27.3 *vs.* 4%) and CUX1 (22.7 *vs.* 0%) in the TTN-MT group were significantly higher than those in the TTN-WT group (P < 0.05).

Conversely, the STK11 mutation frequency (0 *vs.* 24%) in the TTN-MT group was significantly lower than that in the TTN-WT group (P < 0.05). In the TCGA cohort, most driver genes belong to an oncogene, while very few driver genes belong to TSG. We found that the mutation frequencies of most driver genes in the TTN-MT group were significantly higher than those in the TTN-WT group. These frequencies include TP53 (61.9 *vs.* 36.4%), TTN (100 *vs.* 0%), MUC 16 (56.8 *vs.* 25.2%), CSMD 3 (52.9 *vs.* 23%), LRP1B (47.1 *vs.* 20%), USH2A (45.1 *vs.* 18%), ZFHX4 (44.4 *vs.* 17.7%), FLG (35 *vs.* 16.4%), XIRP2 (38.1 *vs.* 13.8%), SPTA1 (33.9 *vs.* 16.4%), NAV3 (30 *vs.* 11.8%), FAT3 (29.6 *vs.* 11.8%), COL11A1 (28.8 *vs.* 12.1%), ZNF536 (29.6 *vs.* 11.1%), ANK2 (28.8 *vs.* 11.1%), CSMD1 (27.2 *vs.* 12.1%), PCLO (30 *vs.* 9.5%), and PCDH15 [29.2 *vs.* 9.8% (**Figures 2B**)]. Additionally, we analyzed and visualized the mutual exclusion and co-occurrence status of the top 20 mutated driver genes in the ICI-cohort and TCGA cohort, respectively (**Figures 2C, D**).

### TIME Under TTN Mutation

**Figure 3:**
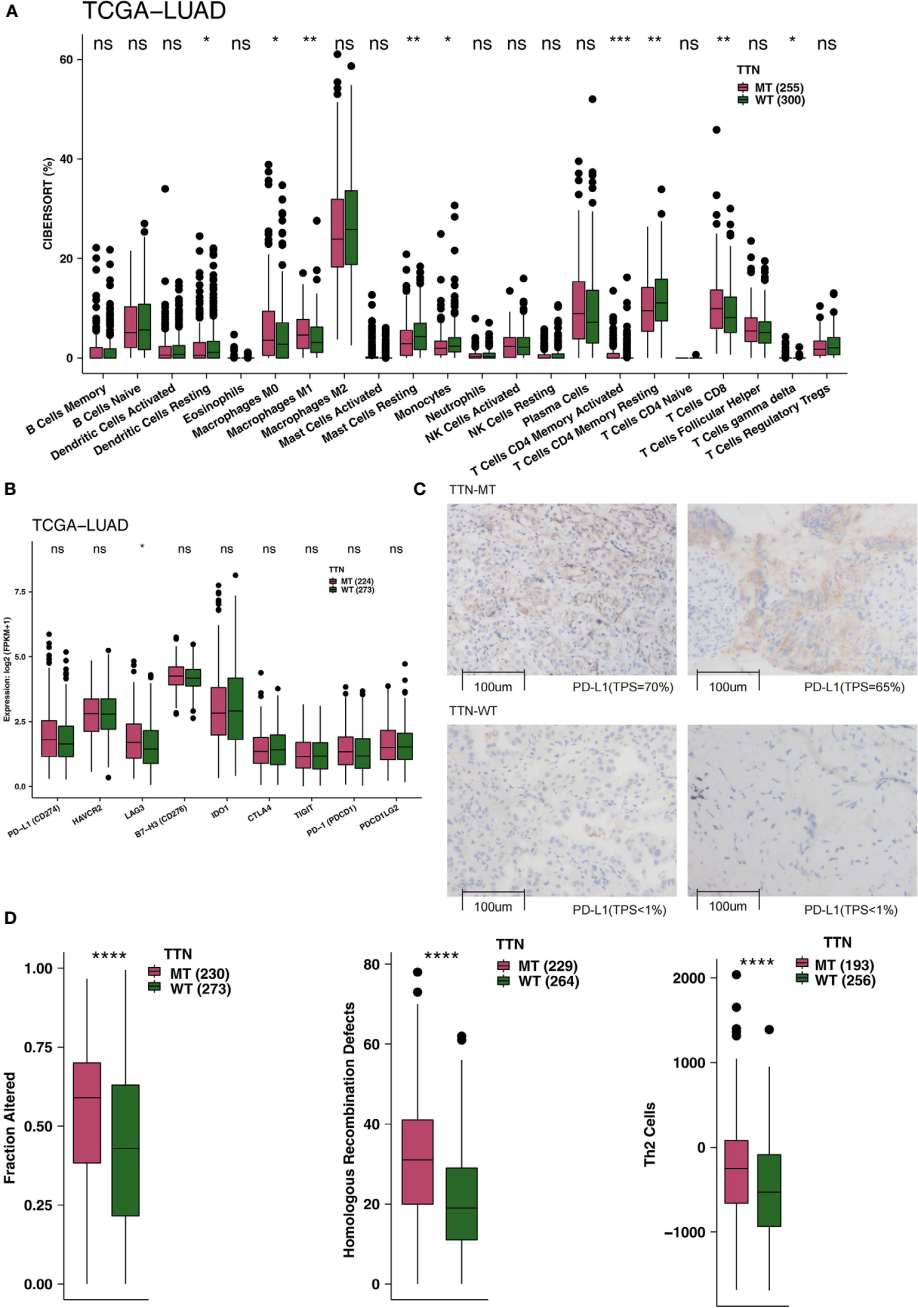
An association of TTN status and immune infiltration in the LUAD patients. **(A)** Comparison of 22 immune cells between TTN-MT and TTN-WT tumors in the TCGA-LUAD cohort. **(B)** Comparison of immune checkpoint molecules between TTN-MT and TTN-WT tumors in the TCGA-LUAD cohort. **(C)** The typical cases for each TPS level between the TTN-MT (two samples; high PD-L1 TPS) and TTN-WT (o samples; no PD-L1 TPS) groups in the Local-LUAD. Using HE- and PD-L1-stained slides, we manually assessed the number of tumor cells, the sample size (diameter), the crush rate with a cutoff value of <1% (no PD-L1 TPS), 1–50% (low PD-L1 TPS), 50%< (high PD-L1 TPS), and the TPS for each biopsy sample using the slide that contained the most tumor cells. The TPS level was evaluated by pathologists who completed training courses in TPS estimation. **(D)** Comparison of immune-related scores between TTN-MT and TTN-WT tumors in the TCGA-LUAD cohort. *P < 0.05; **P < 0.01; ***P < 0.001; ****P < 0.0001, ns, not significant.

TIME is an integral part of cancer patients’ post-immunotherapy response. Consequently, we compared the TIME under different TTN mutation statuses from the perspective of TIME components, such as immune infiltrating cells, examination molecules, and scores. First, CIBERSORT was used to evaluate the relative abundance of 22 immune cell infiltrations in LUAD patients. By comparing the differences in immune cell ratio in different TTN mutation statuses (**Figure 3A**), we found that the M0-type macrophages, M1-type macrophages, activated memory CD4 T cells, and CD8+ T cells were significantly enriched TIME of the TTN-MT group. In contrast, the TIME of the TTN-WT group was enriched with some immune cells with a static function such as resting DCs, resting mast cells, and resting memory CD4 T cells. The results of immune checkpoints showed no significant difference in the expression level of most immune checkpoints between TTN-WT and TTN-MT groups. The expression of LAG3 in the TTN-MT group was significantly higher than that in the TTN-WT group (**Figure 3B**). **Figure 3C** shows typical cases for each TPS level (TTN: two MT *vs.* two WT cases). Additionally, we also compared some immune scores related to immune response, and the results showed that the TTN-MT group was partly altered. The scores of homologous recombination defects and Th2 cells were significantly higher than those of the TTN-WT group (**Figure 3D**).

### TTN-MT Group Has Higher Immunogenicity

**Figure 4:**
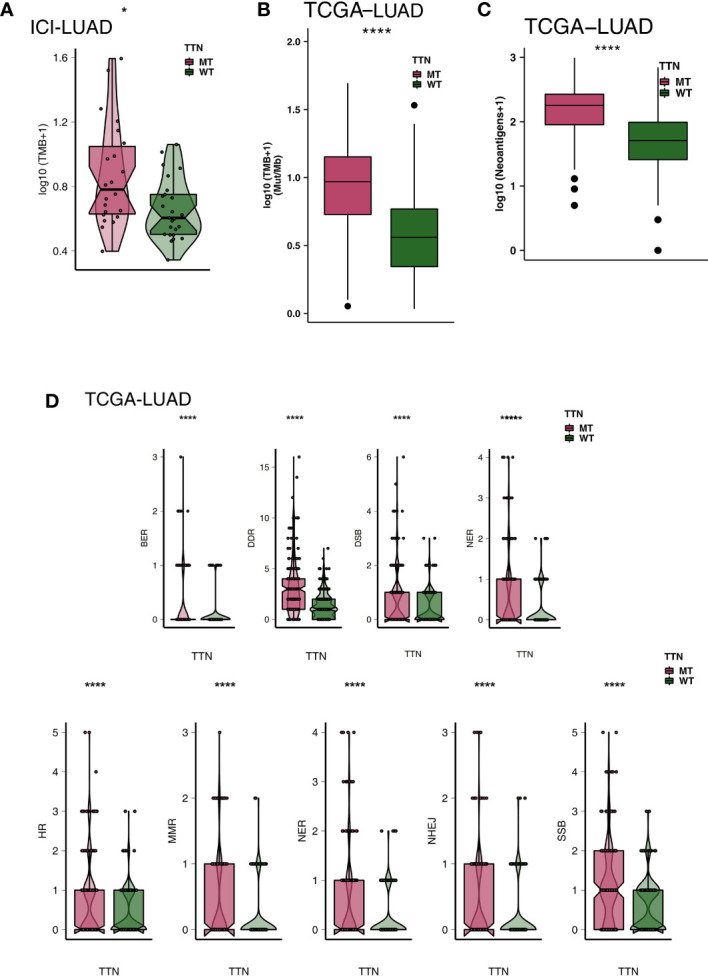
An association of TTN status and immunogenicity in the LUAD patients. Comparison of TMB between TTN-MT and TTN-WT tumors in the ICI-cohort **(A)** and the TCGA-LUAD cohort **(B)**. **(C)** Comparison of NAL between TTN-MT and TTN-WT tumors in the TCGA-LUAD cohort. **(D)** Comparison of mutation counts of DDR signaling pathways between TTN-MT and TTN-WT tumors in the TCGA-LUAD cohort. *P < 0.05; ****P < 0.0001.

High immunogenicity is beneficial for the body in identifying and killing tumors and in producing clinical benefits from immunotherapy. Therefore, to determine immunogenicity, we compared the differences in varying TTN mutation statuses. For TMB, the TTN-MT group had a significantly higher TMB level than the TTN-WT group in both the ICI-cohort and TCGA-LUAD cohort (**Figures 4A, B**; all P < 0.05). Then, we compared NAL in the TCGA cohort, and **Figure 4C** shows that the NAL level of the TTN-MT group was significantly higher than that of the TTN-WT group (**Figure 4C**; P < 0.0001). The DNA damage repair system plays a vital role in correctly repairing DNA damage and preventing excessive accumulation of incorrect DNA. We used eight DDR-related pathways collected from the MsigDB database and merged them into one overall DDR signal. Also, we found that the number of gene mutations in DDR-related pathways in the TTN-MT group was significantly higher than in the TTN-WT group (**Figure 4D**; all P < 0.05).

### Degree of Immune and Pathological Pathway Activation in TTN Mutation Status

**Figure 5:**
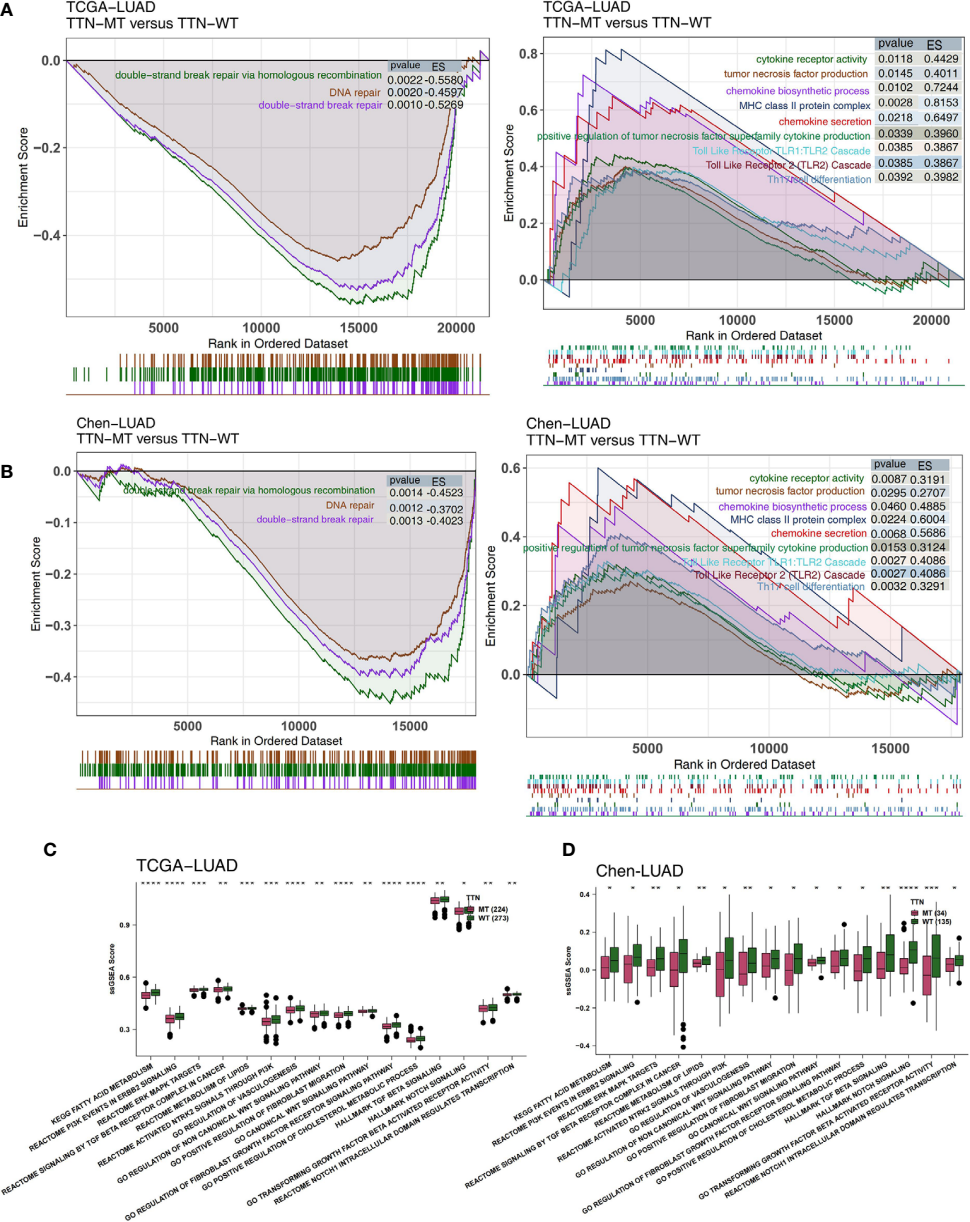
Association of TTN status and the activity of signaling pathways in the LUAD patients. The results of GSEA in the TCGA-LUAD **(A)** and the Chen-LUAD cohort **(B)**. The color of the curve corresponds to the font color of the pathway. The GSEA of hallmark gene sets was downloaded from the MSigDB, and each run was performed with 1,000 permutations. Enrichment results with significant differences between TTN-MT and TTN-WT tumors are shown. Heatmap depicting the mean differences in ssGSEA score between TTN-MT and TTN-WT in the TCGA-LUAD **(C)** and the Chen-LUAD cohort **(D)**. The y-axis indicates the ssGSEA score of pathways. Each square represents the fold change or difference of each indicated ssGSEA score of pathways between TTN-MT and TTN-WT tumors in LUAD. Red indicates upregulation, while blue indicates downregulation. *P < 0.05; **P < 0.01; ***P < 0.001; ****P < 0.0001.

The degree of activation in LUAD patients’ immune- and pathological-related pathways is related to their prognosis of receiving immunotherapy. Therefore, we used GSEA and ssGSEA to evaluate the activity of pathways in LUAD patients. We found that the activity of immune-related pathways in the TTN-MT group was significantly higher than in the TTN-WT group (**Figures 5A, B**). This activity is inclusive of several processes: the chemokine biosynthetic process, chemokine secretary, tumor necrosis factor production, positive regulation of cytokine production in the tumor necrosis factor superfamily, MHC class II protein complex, cytokine receptor activity, Th17 cell differentiation, the Toll-Like Receptor TLR1:TLR2 Cascade, and the Toll-Like Receptor 2 (TLR2) Cascade. In contrast, in the DDR-related pathway (**Figures 5A, B**), the TTN-MT group’s activity was significantly lower than the TTN-WT group in DNA repair, double-strand break repair, double-strand break repair *via* homology recombination, and DNA double-strand break repair.

Additionally, we used the ssGSEA algorithm to evaluate the pathway activity of every LUAD patient. Pathological pathway activity is related to carcinogenesis, and immune depletion in the TTN-MT group was significantly lower than in the TTN-WT group, in both the TCGA (**Figure 5C**) and Chen-LUAD (**Figure 5D**) cohorts. This activity includes canonical Wnt signaling, PI3K signaling, ERK/MAPK signaling, vasculogenesis, and TGF beta.

## Discussion

Immune-system tumor immunotherapy mainly kills tumor cells by activating the body’s antitumor immune function, which has the characteristics of long-lasting curative effects and few adverse reactions (23). In this study, we proved that the non-synonymous mutation status of TTN is related to the high level of immunogenicity, which is mainly manifested in high TMB, high NAL, and the higher mutation level of the DDR pathway. Additionally, we found that LUAD patients with a TTN mutation have an inflammatory TIME characterized by more activated immune cell infiltration and higher immune scores. Most importantly, the mutation status of TTN can be used as an independent predictor of immunotherapy for LUAD patients. This discovery will help clinicians select patients who are more likely to benefit from ICIs in future clinical practice.

Caused by genomic instability, high immunogenicity is especially important in predicting the prognosis of patients receiving immunotherapy (24, 25). As one of the most widely studied markers, many studies have shown that a high level of TMB is closely related to the improved prognosis of patients receiving immunotherapy (25–27). The increased TMB can promote the production of new NAL. DCs can treat and present this new antigen, further mediating the transformation of T cells into mature and activated cytotoxic T cells and finally mediating the antitumor activity of the immune system and enhancing patients’ response to treatment with ICIs (13, 28). Therefore, NAL, another important biomarker, is significantly correlated with the efficacy of immunotherapy (29, 30). Recent research revealed that the DDR-related pathway is another factor that plays an important role in genome stability because it reduces false mutations by repairing incorrect DNA damage (31).

Teo et al. proved that the mutation status of the DDR pathway is related to the improved prognosis of bladder cancer patients being treated with immunotherapy (32). We found that TTN-MT LUAD patients had a higher mutation number in the DDR pathway, and the activity of the DDR-related pathway in their TIME showed a significant downward trend. In this study, we found that LUAD patients with TTN-MT showed higher immunogenicity, such as high TMB, high NAL, and a higher number of non-synonymous mutations in DDR-related pathways. This factor may explain TTN-MT LUAD patients’ improved OS and clinical benefit ratio from immunotherapy.

Along with immunogenicity, studies have shown that inflammatory TIME can also increase patients’ response to immunotherapy (33–35). Reports suggest that CD4+ and CD8+ TILs highly enriched in the tumor are related to a higher response rate after receiving immunotherapy (36, 37). Intratumor tumor-associated macrophages (TAMs) can be reprogrammed under the action of TIME and can be switched between the M1 type for antitumor immunity and the M2 type for promoting tumor proliferation. Invasion of high M1 TAM in the tumor is related to a good prognosis (38). Additionally, in the TIME of TTN-MT, chemokine analysis showed higher activity, TNF secretion. Studies have shown that high TNF levels can promote the secretion of a large number of IFN-γ by effector T cells. This cytokine further upregulates the expression of MHC molecules by activating the STAT1 signaling pathway. Finally, it can prompt the immune system to recognize NALs (39). Additionally, chemokines CXCL9 and CXCL10 can recruit activated T-cells and NK cells and finally trigger their entry into tumors and further exert antitumor activity (40, 41).

In contrast, the activities of some pathological pathways leading to immune depletion are significantly downregulated in TTN-MTs, such as ERK/MAPK, PI3K/Akt, WNT, transforming growth factor-β (TGF-β), FGFR, and lipid metabolism. Williams et al. proved that PI3K inhibitor could alleviate the TIME of immune exhaustion and increase the sensitivity of breast cancer to immune checkpoint blocking treatment (42). Wang et al. discovered that tumor TIME could reverse immunosuppression by blocking abnormally activated PI3K/mTOR signaling (43).

Additionally, the WNT/β signaling pathway plays an important role in forming immune heterogeneity, such as inhibiting T cell activation and infiltration, promoting T cell apoptosis, inhibiting antigen treatment and degree, and inhibiting the killing effect of the immune system on tumor cells (44). TGFβ inhibits tumor infiltration by CXCR3 and CD8+T cells (45). Inhibition of TGFβ is beneficial to the recruitment and infiltration of T cells into the tumor center and further plays a role in antitumor activity (46). Cytokines such as the vascular endothelial growth factor (VEGF), fibroblast growth factor (FGF), and TGFβ can lead to abnormal tumor blood vessels and reduce immune cell infiltration in tumor tissues (47, 48). Cholesterol can further inhibit T cell antitumor activity by binding with the TCRβ transmembrane region and interfering with the TCR signaling pathway (49, 50). These results suggest that the inflammatory TIME in TTN-MT may lead to improved OS time and better clinical benefits for these patients.

However, this research has some limitations. First, we have only explored the relationship between the TTN mutation status and immunotherapy prognosis and response in one COAD cohort of patients treated with ICIs. We still need to verify these results in several cohorts of LUAD receiving ICIs. Second, this study lacks cell and animal experiments for the verification of follow-up mechanisms. We hope that the potential mechanism between TTN mutation and the prognosis of patients receiving immunotherapy can be verified by cell experiments and animal experiments in the future.

## Conclusions

In this study, we found that TTN-MT may be a predictive marker of immunotherapy for LUAD patients. TTN-MT is significantly associated with significantly improved immunotherapy prognoses in LUAD patients who also have significantly increased immunogenicity and inflammatory tumor TIME.

## Data Availability Statement

The original contributions presented in the study are included in the article/**Supplementary Material**. Further inquiries can be directed to the corresponding authors.

## Ethics Statement

Ethical review and approval was not required for the study on human participants in accordance with the local legislation and institutional requirements. Written informed consent for participation was not required for this study in accordance with the national legislation and the institutional requirements.

## Author Contributions

Conceptualization, SL, XY. Formal analysis, ZW. Visualization, ZW. Writing—original draft, CW. Writing—review and editing, SL, XY, ZW, CW. All authors contributed to the article and approved the submitted version.

## Conflict of Interest

The authors declare that the research was conducted in the absence of any commercial or financial relationships that could be construed as a potential conflict of interest.

The reviewer XW declared a shared affiliation, with no collaboration, with the authors, to the handling editor at the time of the review.

## Publisher’s Note

All claims expressed in this article are solely those of the authors and do not necessarily represent those of their affiliated organizations, or those of the publisher, the editors and the reviewers. Any product that may be evaluated in this article, or claim that may be made by its manufacturer, is not guaranteed or endorsed by the publisher.

## Supplementary Material

Supplementary Table 1 The Fisher’s exact test result of the Top 20 mutated driver genes between TTN-MT and TTN-WT tumors in the ICI-cohort.

Supplementary Table 2 The Fisher’s exact test result of the Top 20 mutated driver genes between TTN-MT and TTN-WT tumors in the TCGA-LUAD cohort.

Supplementary Table 3 The co-occurrence (or mutually exclusive relation) results between pairs of mutated driver genes in the ICI cohort.

Supplementary Table 4 The co-occurrence (or mutually exclusive relation) results between pairs of mutated driver genes in the TCGA-LUAD cohort.
