# Supplementary material for: Effect of TTN Mutations on Immune Microenvironment and Efficacy of Immunotherapy in Lung Adenocarcinoma Patients
Source: Front Oncol. 2021 Aug 26;11:725292. doi: 10.3389/fonc.2021.725292 (PMC8426356; doi:10.3389/fonc.2021.725292)
Supplement: Supplementary file 5 [file DataSheet_5.docx]

Pathological diagnosis

Pathological diagnosis was conducted by pathologist using hematoxylineosin (HE) stained slides. After the diagnosis of LUAD, PD-L1 immunohistochemistry staining of each biopsy sample was conducted and assessed by at least two pathologists. The samples, which were formalin-fixed and paraffin-embedded, were sliced at a thickness of 4 μm. The sections were processed for 20 min at 97°C for deparaffinized and inactivating enzymes. Sequentially, the samples were stained for PD-L1 with an anti-human PD-L1 antibody. PD-L1 expression was evaluated in our institution using companion diagnostic PD-L1 immunohistochemistry (IHC) (PD-L1 IHC 22C3, pharmDx, Dako/Agilent, Santa Clara, United States) with autostainer Link 48, detecting driver mutation in parallel. Following the standard recommendation of previous publication[1] PD-L1 protein expression was determined by tumor proportion score (TPS).

References

1. Li, H.; Durbin, R. Fast and accurate short read alignment with Burrows-Wheeler transform. *Bioinformatics* **2009**, *25*, 1754–1760, doi:10.1093/bioinformatics/btp324.
